# Editorial: The Importance of Genetic Literacy and Education in Medicine

**DOI:** 10.3389/fgene.2022.910530

**Published:** 2022-04-28

**Authors:** Nina Pereza, Borut Peterlin, Saša Ostojić, Željka Poslon

**Affiliations:** ^1^ Centre for Genetic Education, Faculty of Medicine, University of Rijeka, Rijeka, Croatia; ^2^ Department of Medical Biology and Genetics, Faculty of Medicine, University of Rijeka, Rijeka, Croatia; ^3^ Clinical Institute of Genomic Medicine, University Medical Centre Ljubljana, Ljubljana, Slovenia

**Keywords:** genetic literacy, genetic education, medical education, medical genetics, medicine

Genetic literacy is a critical prerequisite for appropriate care for patients with genetic disorders, and includes the literacy on basic concepts in human and medical genetics. Medical genetics is one of the fastest-developing medical specializations, and advances in the development of new, comprehensive genetic and genomic testing methods are becoming increasingly integrated into various parts of medicine. Unfortunately, these advances have not been accompanied by an adequate level of genetic literacy in medical students, non-genetic health professionals involved in the care of patients with genetic disorders, as well as general public, including patients. Consequently, the demands for appropriate, needs-based genetic education on all levels are increasing.

The focus of this Research Topic includes the state of the current level of genetic literacy among medical students, non-genetic health professionals, patients and general public, as well as the current state of activities, options and future directions for genetic education in these groups. Additionally, we addressed the needs and possibilities of genetic education for patients with rare diseases. The Research Topic comprises 10 articles, with as much as 59 eminent authors from 20 countries.

The Research Topic begins with a methods article by Tobias et al., who emphasize the concerns that the current COVID-19 pandemic has raised in individuals with genetic disorders regarding both the viral infection and its specific implications and advisable precautions. These concerns were discussed on the ScotGEN Steering Committee and the Education Committee of the European Society of Human Genetics. Consequently, an up-to-date online hub of genetics-related COVID-19 information resources was created and provided freely online at www.scotgen.org.uk and www.eurogems.org.

Sassano et al. in their original research article summarized the educational initiatives aimed at increasing citizens’ literacy in omics sciences worldwide, performing a web search. They identified a variety of initiatives aimed at improving citizens’ literacy in omics sciences, with the largest majority carried out in the United States and being web-based. Their results showed heterogeneity among the initiatives as to the dealt topics and the adopted methods.

Considering that the care for patients with rare diseases requires a multidisciplinary approach, Domaradzki and Walkowiak performed an original research, assessing the awareness of rare diseases among nursing, physiotherapy and medical students in Poland using a questionnaire. Although 98% of respondents had heard of the term “rare disease,” most students had problems in defining their most common causes and prevalence. Almost 92% of medical students, and 84% of physiotherapy and nursing students did not feel prepared for caring for these patients. The results emphasize the need for better education in this field.

The rewiew article by Liehr summarizes the general background on non-invasive prenatal testing (NIPT), differences of NIPT platforms, advantages and limitations of NIPT, as well as consequences of insufficient counselling before and after NIPT. Unfortunately, gynecologists and obstetricians who discuss the use of NIPT with patients may lack specific training on the interpretation of results, although they have a highly qualified background in their specialty area(s). The author emphasizes the importance that the corresponding scientific societies close the potential knowledge gaps quickly and comprehensively to ensure optimal patient care.

In their mini review, Little and Gunter discuss the current literature describing genetic literacy and genetic testing rates for autism spectrum disorders (ASD). They conclude that the current level of the population’s genetic literacy is insufficient to ensure the individuals’ informed decisions about their genetic information. In addition, only 22% of families undergo genetic testing after diagnosis. Therefore, the authors suggest that improving genetic literacy in ASD populations can also improve attitudes toward genetic testing.

Zimani et al. provide a mini review regarding the current state of educational activities within national genomic projects for different target groups and identify good practices that could contribute to patient empowerment, public engagement, proficient healthcare professionals, and lend support to personalized medicine. The authors reviewed 41 current national genomic projects and identified 16 projects specifically describing the approach to genomic education. Hopefully, the initial efforts made by national genomic projects will result in durable national solutions leading to further implementation of personalized medicine in healthcare systems.

An interesting perspective article by Tobias et al. summarizes how the European Society of Human Genetics is adapting to deliver innovative genetic educational activity. The Society works through many approaches, including educational sessions at the annual conference; training courses in general and specialist areas of genetics; an online resource of educational materials (EuroGEMS); and a mentorship scheme. Their Education Committee is implementing new approaches to expand the reach of its educational activities and portfolio.

In their brief research report, Majstorović et al. evaluate current genomics content in the curriculum of undergraduate and graduate nursing studies programs in Croatia in 2020/2021, and measure the genomic literacy through assessing participants’ understanding of genomic concepts critical to nursing practice. Their results indicate that the current genomics content is inadequate and dis-concordant among universities. Moreover, genomic literacy of nursing students was low. The authors emphasize that the curricula for undergraduate and graduate nursing studies programs needs revision and implementation of modern genomics education.

In the original research article by Vidgen et al. a training session, introducing Health Interpreters to genetics was developed and evaluated. The online training was delivered multiple times as a single 2-h session comprising lectures and activities. Participants completed questionnaires to assess the impact of training on knowledge, attitude, self-efficacy, and self-reported practice behaviour. The results show that most respondents and Health Interpreters agreed that the training was useful and acceptable. Increased delivery of training and associated research is needed to assess findings in a larger cohort and to measure the impact on patients.

Finally, Pereza et al. in their original research article perform the first research on the current state of compulsory basic and clinical courses in genetics for medical students offered at medical faculties in six Balkan countries with Slavic languages (Bosnia and Herzegovina, Croatia, Montenegro, North Macedonia, Serbia, and Slovenia). Except for Slovenia, all other countries offer either courses in basic education in human genetics or both basic education in human genetics and clinical education in medical genetics. Unfortunately, due to huge differences in course designs, the authors emphasize the need for future collaboration in reaching a consensus on medical genetics education in Balkan countries.

